# Supplementary material for: Experience-dependent reconfiguration of receptors at a sensory compartment regulates neuronal plasticity
Source: bioRxiv. 2025 Aug 13:2025.08.13.670147. Preprint. [Version 1] doi: 10.1101/2025.08.13.670147 (PMC12377508; doi:10.1101/2025.08.13.670147)
Supplement: 1 [file NIHPP2025.08.13.670147V1-supplement-1.pdf]

bioRxiv preprint doi: https://doi.org/10.1101/2025.08.13.670147; this version posted August 13, 2025. The copyright holder for this preprint (which was not certified by peer review) is the author/funder, who has granted bioRxiv a license to display the preprint in perpetuity. It is made available under aCC-BY 4.0 International license.

SUPPLEMENTARY FIGURE LEGENDS

Supplementary Figure 1. Analysis of temperature-dependent localization of GCY-18 and GCY-23 in the AFD sensory compartment. Related to Figure 1.

A) (Left) Representative images of GCY-18::GFP localization in the AFD sensory endings in the indicated temperature conditions. Scale bar: 2 µm. (Right) Average intensity distributions of GCY-18::GFP in the AFD sensory compartment from line scan analyses (indicated by red horizontal line in images at left). The percentage of animals exhibiting relocalization of GCY-18::GFP is indicated. n=40-47 sensory endings each; at least 2 independent experiments.

B) (Left) Summary of previous findings that *pyt-1* is transcriptionally upregulated by temperature stimuli and the CREB transcription factor[1]. (Right) Representative images of PYT-1::GFP localization in the AFD sensory ending before (0 hours) and after (4 hours) a shift from 15°C to 25°C.

C) Representative images of GCY-23::TagRFP localization in the AFD sensory endings in the indicated temperature conditions. Scale bar: 2 µm. The percentage of animals exhibiting relocalization of GCY-23::TagRFP is indicated. Representative images are from the data set shown in D.

D) Representative images (left) and quantification (right) of GCY-23::TagRFP levels in the AFD sensory endings of adult wild-type and *pyt-1* mutant animals at the indicated temperature conditions. Each dot is the measurement from a single AFD sensory ending. Scale bar: 10 µm. n=25-27 sensory endings; 2 independent experiments. Since independent experiments were performed using different magnification objectives, data are normalized to the control mean values at 15°C for the relevant day. Horizontal and vertical lines indicate mean and SD, respectively. ***: different at $p<0.001$ (t test), ns – not significant.

bioRxiv preprint doi: https://doi.org/10.1101/2025.08.13.670147; this version posted August 13, 2025. The copyright holder for this preprint (which was not certified by peer review) is the author/funder, who has granted bioRxiv a license to display the preprint in perpetuity. It is made available under aCC-BY 4.0 International license.

Supplementary Figure 2. Endosomal localization of PYT-1 and GCY-23. Related to Figure 2.

A) Representative images of GCY-23::GFP and TagRFP::RAB-5 localization in the absence of a temperature shift (after cultivation at 15°C). Scale bar: 2 µm.

B) Representative images of GCY-23::GFP and TagRFP::RAB-5 localization in *pyt-1(oy160)* null mutants after a shift from 15°C to 25°C for 4 hours. Scale bar: 2 µm.

Supplementary Figure 3. Analysis of dendritic trafficking of GCY-18 and GCY-23. Related to Figure 2.

A, B) Representative kymographs of GCY-23::GFP (A) and GCY-18::GFP (B) movement in the AFD dendrite of a wild-type and *pyt-1(oy160)* null mutant animal in the absence of a temperature shift (after cultivation at 15°C - 0 hours) and after 4 hours of temperature shift from 15°C to 25°C (4 hours).

C) Representative kymographs of GCY-23::GFP movement in the AFD dendrite of a wild-type and *rab-5(S33N)* mutant animal after 4 hours of temperature shift from 15°C to 25°C.

Tracks moving from the sensory ending (SE) to the cell body (CB) represent retrogradely moving cargo. Tracks moving from the CB to the SE represent anterogradely moving cargo.

Supplementary Figure 4. Role of PY motifs in PYT-1 function. Related to Figure 3.

A) Alignment of the C-terminal amino acid sequences of *C. elegans* PYT-1 and orthologous proteins in other nematodes. PY motifs are marked in red text.

bioRxiv preprint doi: https://doi.org/10.1101/2025.08.13.670147; this version posted August 13, 2025. The copyright holder for this preprint (which was not certified by peer review) is the author/funder, who has granted bioRxiv a license to display the preprint in perpetuity. It is made available under aCC-BY 4.0 International license.

B) Representative kymographs of GCY-23::GFP movement in the AFD dendrite in a wildtype and *pyt-1(3xPY)* mutant animal in the absence of a temperature shift after cultivation at 15°C (0 hours) and after 4 hours of temperature shift from 15°C to 25°C (4 hours). Tracks moving from the sensory ending (SE) to the cell body (CB) represent retrogradely moving cargo. Tracks moving from the CB to the SE represent anterogradely moving cargo.

C) (Left) GCaMP traces from AFD in animals of the indicated genotypes before and after a temperature upshift in response to a temperature ramp (green line). Thick lines and shading: average ΔF/F change and SEM, respectively. Dashed vertical lines: T^*_{AFD} for the indicated genotypes. (Right) Quantification of T^*_{AFD} in animals of the indicated genotypes calculated from traces at left. Each dot is a measurement from a single animal. n=22-24 animals each; 2 independent experiments. Horizontal and vertical lines indicate mean and SD, respectively. ns – not significant.

Movie S1. Representative movie showing movement of GCY-23::GFP-containing particles in the distal AFD dendrite in a wild-type animal at 15°C.

Movie S2. Representative movie showing movement of GCY-23::GFP-containing particles in the distal AFD dendrite in a wild-type animal following a temperature upshift from 15°C to 25°C for 4 hours.

Movie S3. Representative movie showing movement of GCY-18::GFP-containing particles in the distal AFD dendrite in a wild-type animal at 15°C.

Movie S4. Representative movie showing movement of GCY-18::GFP-containing particles in the distal AFD dendrite in a wild-type animal following a temperature upshift from 15°C to 25°C for 4 hours.

bioRxiv preprint doi: https://doi.org/10.1101/2025.08.13.670147; this version posted August 13, 2025. The copyright holder for this preprint (which was not certified by peer review) is the author/funder, who has granted bioRxiv a license to display the preprint in perpetuity. It is made available under aCC-BY 4.0 International license.

Movie S5. Representative movie showing movement of GCY-23::GFP-containing particles in the distal AFD dendrite in a *pyt-1(oy160)* animal at 15°C.

Movie S6. Representative movie showing movement of GCY-23::GFP-containing particles in the distal AFD dendrite in a *pyt-1(oy160)* animal following a temperature upshift from 15°C to 25°C for 4 hours.

Movie S7. Representative movie showing movement of GCY-18::GFP-containing particles in the distal AFD dendrite in a *pyt-1(oy160)* animal at 15°C.

Movie S8. Representative movie showing movement of GCY-18::GFP-containing particles in the distal AFD dendrite in a *pyt-1(oy160)* animal following a temperature upshift from 15°C to 25°C for 4 hours.

SE: sensory ending, CB: cell body.

REFERENCES

1. Harris, N., Bates, S.G., Zhuang, Z., Bernstein, M., Stonemetz, J.M., Hill, T.J., Yu, Y.V., Calarco, J.A., and Sengupta, P. (2023). Molecular encoding of stimulus features in a single sensory neuron type enables neuronal and behavioral plasticity. Curr Biol *33*, 1487-1501.e7. https://doi.org/10.1016/j.cub.2023.02.073.

bioRxiv preprint doi: https://doi.org/10.1101/2025.08.13.670147; this version posted August 13, 2025. The copyright holder for this preprint (which was not certified by peer review) is the author/funder, who has granted bioRxiv a license to display the preprint in perpetuity. It is made available under aCC-BY 4.0 International license.

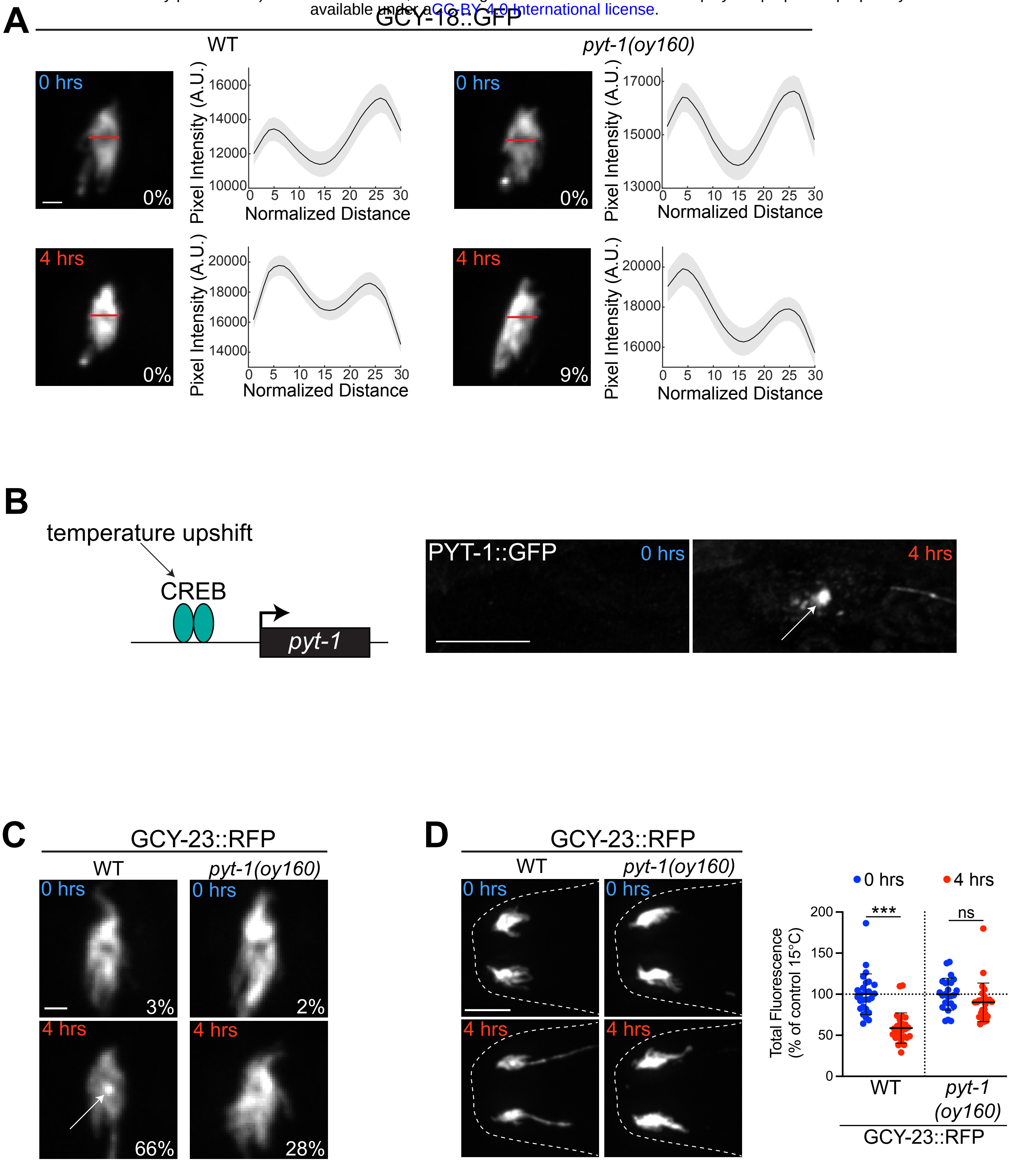

Harris, Dutta et al., Supplementary Figure 1

bioRxiv preprint doi: https://doi.org/10.1101/2025.08.13.670147; this version posted August 13, 2025. The copyright holder for this preprint (which was not certified by peer review) is the author/funder, who has granted bioRxiv a license to display the preprint in perpetuity. It is made available under aCC-BY 4.0 International license.

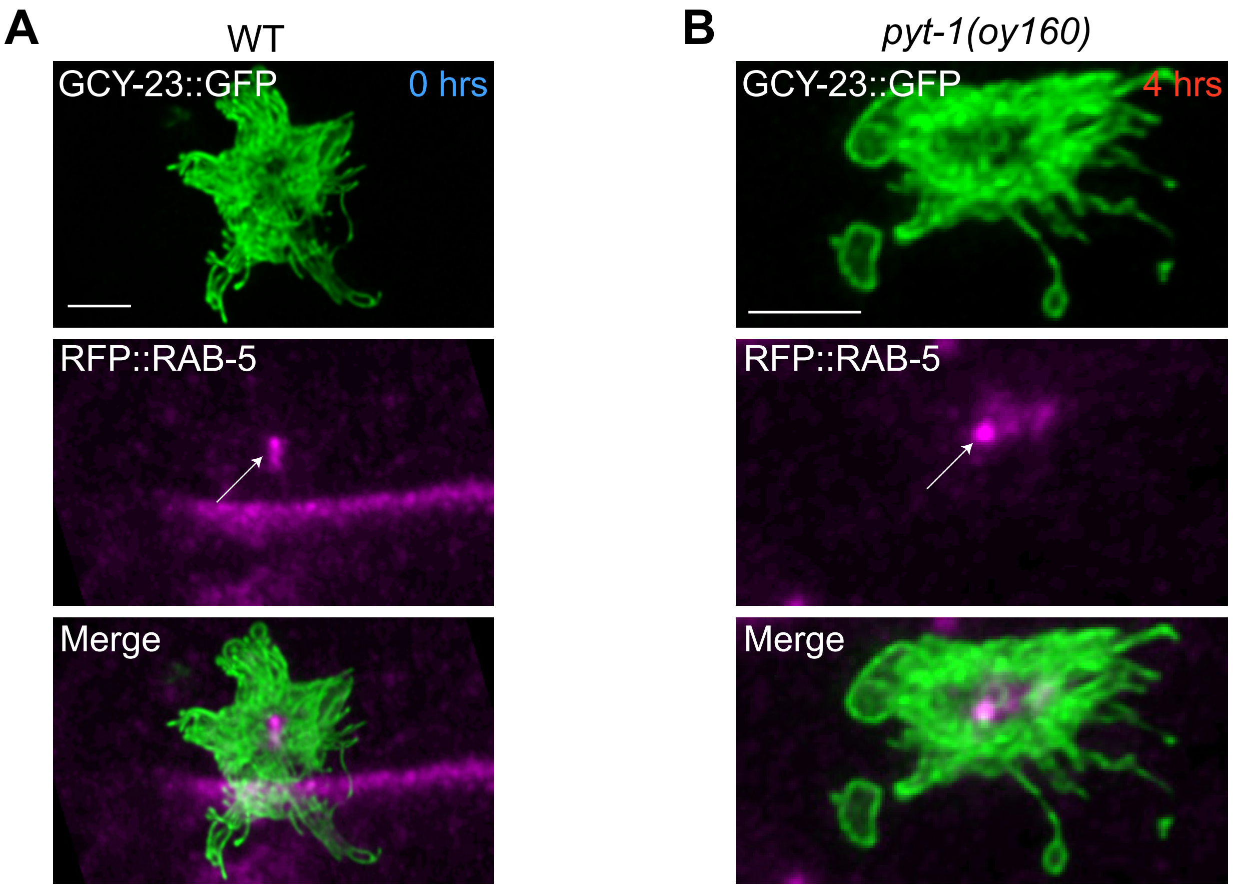

Harris, Dutta et al., Supplementary Figure 2

bioRxiv preprint doi: https://doi.org/10.1101/2025.08.13.670147; this version posted August 13, 2025. The copyright holder for this preprint (which was not certified by peer review) is the author/funder, who has granted bioRxiv a license to display the preprint in perpetuity. It is made available under aCC-BY 4.0 International license.

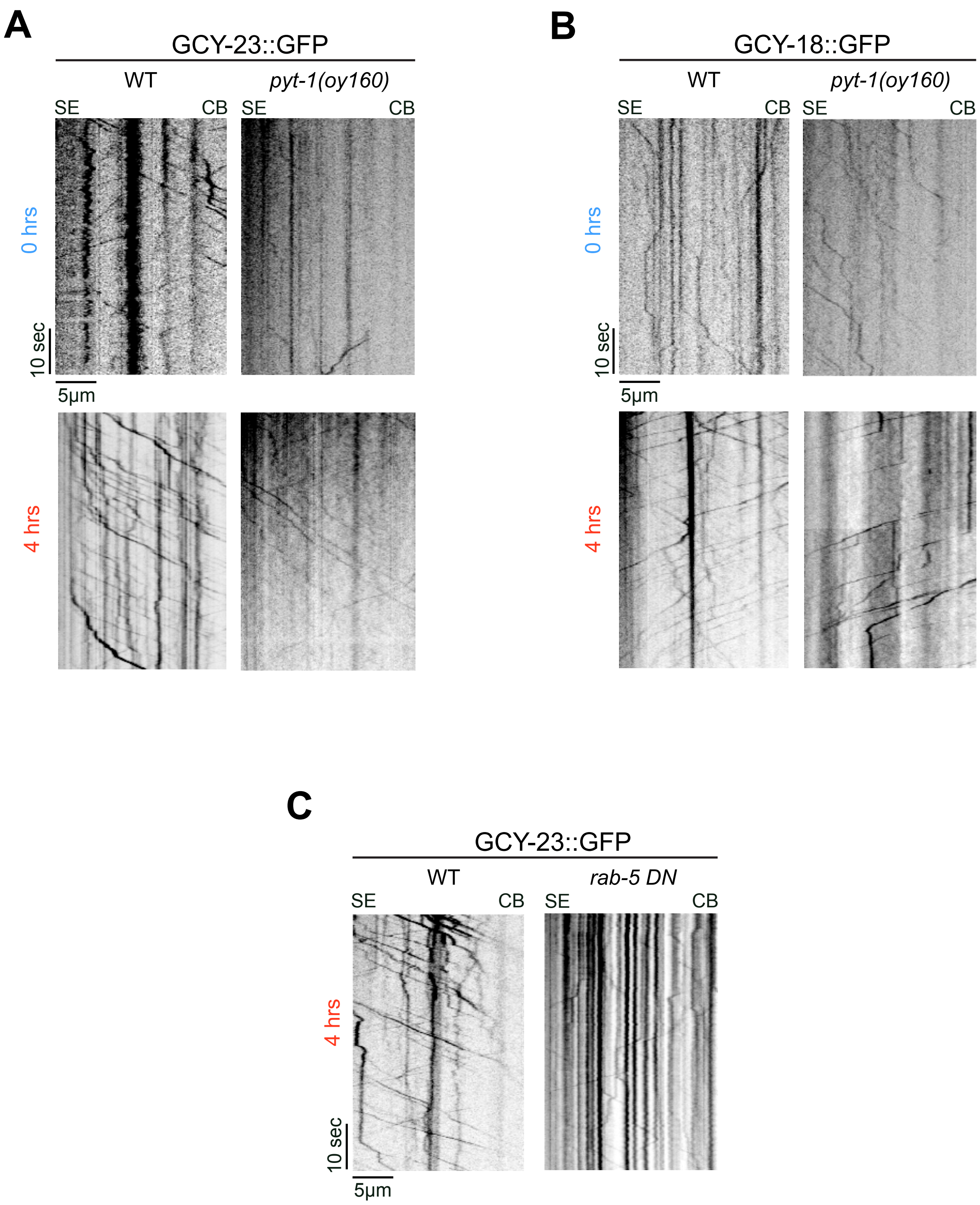

Harris, Dutta et al., Supplementary Figure 3

bioRxiv preprint doi: https://doi.org/10.1101/2025.08.13.670147; this version posted August 13, 2025. The copyright holder for this preprint (which was not certified by peer review) is the author/funder, who has granted bioRxiv a license to display the preprint in perpetuity. It is made available under aCC-BY 4.0 International license.

A

PYT-1 C-term

C. elegans	VEIEVPYYSSVNMPQILLSSE-EHPPEYY--ELESARASPLPSYDDVMYCDQLNRSFQNLLSAR
C. nigoni	LEIELPYYTNVDVPRILLTSE-ELPPDYL--ELESARASPLPSYDDVMYCDQLNRSFQNLLSAR
C. sinica	VEIELPYYSSLNVPQIMLSSE-ELPPEYH--ELESARASPLPSYDDVMYCDQLNRSFQNLLSAR
C. quiockensis	-DIDIPYYSSSNLPQIMLENE-ELPPEYF--ELESARASPLPSYDDVMYCDQLNRSFQNLFSPR
C. tropicalis	VEMELPYYSSTNVPQIILSSD-ELPPEYH--ELESARASPLPSYDDVVYCDQLNRSFQNLLSAR
C. tribulationis	AEMEIPYYSSINVPQIMLSSE-ELPPEYH--ELESARASPLPSYDDVMYCDQLNRSFQNLLSPR
C. japonica	MDLDVLHYTASSLPQIMLQPE-ELPPEYN--ELESARASPLPSYDDVMYCDQLNRSFQNLLSAR
C. becei	MEVEVPYFSSVNLPQIMLEPE-ELPPEYH--ELESARASPLPSYDDVMYCDQLSRSFQNLLSAR
C. remanei	CEMELPYYSVLNIPKILLTPE-ELPPEYH--ELESARASPLPSYDDVMYCDQLSRSFQNLLSPR
C. panamensis	MDVEMPYYSTVSLPQIMLEPE-ELPPEYH--ELESARASPLPSYDDVMYCDQLNRSFQNLLSAR
C. briggsae	IDIELPYYTNVNVPRILLTSE-ELPPDYH--ELESARASPLPSYDDVMYCDQLNRSFQNLLSAR
C. sulstoni	-DIELPFYSSSNVPQILLQPE-ELPPEYY--ELESARASPLPSYDDVMYCDQLNRSFQNLLSAR
C. waitukubuli	MDAELPYFS-VNIPQILLEPE-EPPPEYH--ELESARASPLPSYDDVMYCDQLNRSFQNLLSAR
C. angaria	--LDIPYYTSHNLPSIMLENE-ELPPEYF--ELESARASPLPSYDDVMYCDQLNRSFQNLISAR
C. auriculariae	-ELESIVLSTYNLPQICQFSD-DEPPEYP--HDGSARASPIPTYDEVIYCDQLIRSFEGLLPVQ
C. uteleia	-EIEVPYYSVYNLPKIMLESE-ELPPEYY--DLESARASPLPSYDEMIYCDQLSRSFQNLLSAR
C. bovis	VEMDVPLYSTQNLPQIMLANE-ELPPEYA--ELESARASPLPSYDDVMYCEQLNRSFQNLLSAT
D. pachys	TSYEPPMYTTTNPPQIMCEIE-GDLPEYF----ECGRASPLPSYDDIIYHDRLCSSMQNLLA--
D. coronatus	TGYEPPMYTTRNPPLIMCEIE-GDPPEYF----ECGRASPLPSYNDIIYHDRLCSSMQNLLA--
A. caninum	GSCDIAILIPEPIPELMYQCP-SDPPEYS--E--AARASPLPSYDDVIYCDQVARSFQNLISVK
A. ceylanicum	GSCDIAILIPEPIPELMYQCP-SDPPEYS--E--AARASPLPSYDDVIYCDQVARSFQNLISVK
C. parvicauda	LESQMPLYTIDNPPMVFLKRE-DEPPEYEDCELALARSSPLPSYEDAIYYEQQNRSFQNLLSA-
M. belari	IDDIKAVYQVNGPPMILYENECDELPDYP--NSQTRSASPLPSYDDVIYCDQVNRSFQNLLSTV
M. spiculigera	GADVKNVFHVTQPPRILYENE-EELPDYP--DYGSRSASPLPSYDDVVYCDQVNRSFQNLLNVV

B

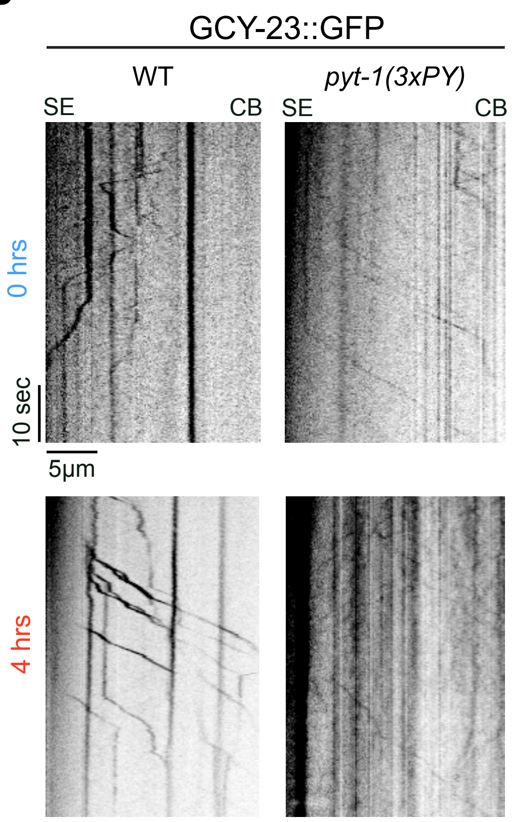

C

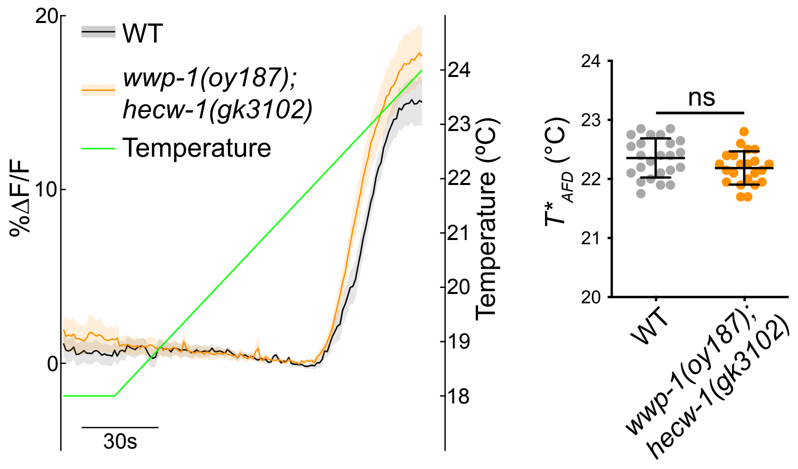

Harris, Dutta et al., Supplementary Figure 4
